# Exploring *Inductive Linearization* for simulation and estimation with an application to the Michaelis–Menten model

**DOI:** 10.1007/s10928-022-09813-z

**Published:** 2022-07-05

**Authors:** Sepideh Sharif, Chihiro Hasegawa, Stephen B. Duffull

**Affiliations:** Otago Pharmacometrics Group, School of Pharmacy, University of Otago, Dunedin, New Zealand

**Keywords:** Nonlinear ordinary differential equations, (PKPD) model development, Numerical methods, Optimization, *Inductive Linearization*, Adaptive step size algorithm

## Abstract

Nonlinear ordinary differential equations (ODEs) are common in pharmacokinetic–pharmacodynamic systems. Although their exact solutions cannot generally be determined via algebraic methods, their rapid and accurate solutions are desirable. Thus, numerical methods have a critical role. *Inductive Linearization* was proposed as a method to solve systems of nonlinear ODEs. It is an iterative approach that converts a nonlinear ODE into a linear time-varying (LTV) ODE, for which a range of standard integration techniques can then be used to solve (e.g., *eigenvalue decomposition* [EVD]). This study explores the properties of *Inductive Linearization* when coupled with EVD for integration of the LTV ODE and illustrates how the efficiency of the method can be improved. Improvements were based on three approaches, (1) incorporation of a convergence criterion for the iterative linearization process (for simulation and estimation), (2) creating more efficient step sizes for EVD (for simulation and estimation), and (3) updating the initial conditions of the *Inductive Linearization* (for estimation). The performance of these improvements were evaluated using single subject stochastic simulation-estimation with an application to a simple pharmacokinetic model with Michaelis–Menten elimination. The reference comparison was a standard non-stiff Runge–Kutta method with variable step size (*ode45*, MATLAB). Each of the approaches improved the speed of the *Inductive Linearization* technique without diminishing accuracy which, in this simple case, was faster than *ode45* with comparable accuracy in the parameter estimates. The methods described here can easily be implemented in standard software programme such as R or MATLAB. Further work is needed to explore this technique for estimation in a population approach setting.

**Supplementary Information:**

The online version contains supplementary material available at 10.1007/s10928-022-09813-z.

## Introduction

The term nonlinearity is used to describe the relationship between two variables that is other than linear. However, its interpretation depends on the discipline. For example, in pharmacokinetics, linearity implies that the relationship between concentration and dose is linear (i.e., proportional). Hence, this departure signifies nonlinear pharmacokinetics (despite even linear PK models being statistically nonlinear). In pharmacodynamics, the linearity depicts the relationship between concentration and effect or subsequent bio-measures in the following effect cascade. These definitions are also readily represented as ordinary differential equations (ODEs), where nonlinear PK or PKPD models typically yield nonlinear ODEs.

Solving systems of nonlinear differential equations is therefore of significant interest in applying pharmacokinetic-pharmacodynamic models and in particular systems pharmacology models. Ideally, analytical solutions are desired since they provide a clear and direct path to explore the underlying mechanisms and reveal core relationships between different model components. However, finding closed-form solutions to these nonlinear systems is uncommon except in exceptional cases. For example, using the transcendent *X* function family and the limit case of the *Lambert W* function has been described as a solution to the Michaelis–Menten PK model when considering intravenous (rather than extravascular) dosing [1]. In contrast, standard numerical techniques such as Runge–Kutta and LSODA are effective solutions and can be applied to a wide range of linear or nonlinear ODEs. These methods, however, involve a local search for the solution at the current time step. These numerical solutions are available only at discrete time points and are blind to the solutions earlier or later in time. Furthermore, like all numerical solvers, they may require user input in relation to the stiffness of the ODEs and are potentially subject to issues of singularities [2] or multiple solutions [3]. Typically we also find that run-times are affected by the degree of nonlinearity of the problem.

*Inductive Linearization* is a numerical solver [4] that has been developed for generating approximated solutions to nonlinear systems based on iterative linearization to yield a linear time-varying (LTV) ODE [5]. The method is then paired with an integrator to solve the LTV system. Analytical, closed-form solutions exist for simple systems of LTV ODEs [6–8]. For more complicated systems, a range of integration techniques can be used. In this work, we pair *Inductive Linearization* (IndLin) with *eigenvalue decomposition* (EVD) for integration using matrix exponentials (similar to [5]). Although the method has been examined previously [5], this study is unique in that it applies the optimized method to parameter estimation. The core concept of the two properties is explained here to illustrate how the method can solve nonlinear ODEs in a PKPD framework. The standard form of a nonlinear ODE is defined as:

1 $$\frac{dy}{dt}=f\left(t,y\right)+A\left(t,y\right)y;\quad y\left({t}_{0}\right)={y}_{0}$$

where $t$ is time, $y(t)={{(y}_{1}\left(t\right),\dots ,{y}_{m}(t))}^{T}$ is an $m \times 1$ vector of dependent variables (i.e., $m$ is the number of the states in the PKPD system). Both $f$ and $A$ are functions that may be a matrix of parameters or have some more complicated functional forms, and ${y}_{0}$ is the initial condition for the differential equation. In the following notation we simplify the system such that $f$ is no longer dependent on $y$ (note this is not a requirement and is for simplicity of notation only). The system can therefore be linearized to yield (Eq. 2)

2 $$\frac{d{y}^{[n]}}{dt}=f(t)+A\left(t,{y}^{\left[n-1\right]}\right){y}^{\left[n\right]}; \quad y\left({t}_{0}\right)={y}_{0}$$

Here, ${y}^{\left[n-1\right]}(t)$ is a vector of known response variable values that arise from the previous iteration. The initial plug-in values for ${y}^{\left[n=0\right]}\left(t\right)$ as the starting point for *Inductive Linearization* are set to 0 for all time points*.* Then as the number of iterations of the linearization $n$ approaches $\infty ,$ the solution to Eq. 2 will yield an exact LTV solution to Eq. 1 with the solution being available for any$t\in [0, T]$, where $T$ is the upper bound of the integration time interval of interest. Choosing a maximum value of$n$, denoted$N$, is a critical step in determining the accuracy of the linearization method. We then apply EVD to integrate the LTV system in Eq. 2 [5], which will provide an integrated solution on the interval $[0, T]$.

This study explores improvements in the efficiency of IndLin coupled with EVD integration as a solution to a simple Michaelis–Menten example using stochastic simulation and estimation. This will be compared with a reference numerical ODE solver (*ode45*, MATLAB, with Absolute Tol = $1e-6$ and Relative Tol = $1e-3$ for all examples in this work). This work is divided into six sections. (2) An application of IndLin to the Michaelis–Menten example, (3) improvement in the efficiency of IndLin coupled with EVD when applied to simulation, (4) improvement in the efficiency of IndLin-EVD when applied to estimation, (5) an evaluation of IndLin-EVD based using SSE methods and, (6) the inference and limitations of the applied methods are described. The system in this example is not stiff, however this feature of IndLin has been studied elsewhere [5].

## Application of *Inductive Linearization* to the Michaelis–Menten example

Nonlinearity is often seen in metabolic processes. The most common expression is the Michaelis–Menten equation. A general form for a drug administered extravascularly and assuming a first-order input from a depot site is given (Eq. 3).

3 $$\frac{dC}{dt}=\frac{{k}_{a}D{e}^{{-k}_{a}t}}{V}-\frac{{V}_{max}/V}{{K}_{m}+C}C; \quad C\left({t}_{0}\right)=0;$$

where $C\left({t}_{0}\right)$ is the initial drug concentration in the blood (at time zero) with units of $mg/L$, $D$ is dose (a single dose of $3 mg$ in our example). The parameters $V$ with units of $L$ is the apparent volume of distribution, ${k}_{a}$ (first-order absorption rate constant with units$/h$), ${V}_{max}$ (maximum velocity of the enzyme, units $mg/h$), and ${K}_{m}$ (Michaelis–Menten constant – defined as the concentration $\left(mg/L\right)$ for which the enzyme is at the half-maximal rate).

The parameters and their values are given in Table 1. Application of IndLin for the same pharmacokinetic example using a different integrator method has been described elsewhere [4]. Here, our integrator method is EVD.

**Table 1:** Parameter values for the Michaelis–Menten example (Eq. 3)

Parameters	Units	Values
${k}_{a}$	$/h$	$1$
${K}_{m}$	$mg/L$	$0.5$
${V}_{max}$	$mg/h$	$0.2$
$V$	$L$	$1$
$\sigma$*		$0.1$
${\omega }_{{k}_{a}}^{2}$		$0.1$
${\omega }_{{K}_{m}}^{2}$		$0.1$
${\omega }_{{V}_{max}}^{2}$		$0.1$
${\omega }_{V}^{2}$		$0.1$

*Exponential error

This system can be written in the form (Eq. 1) as

4 $$A\left(t,C\right)=-\frac{{V}_{max}/V}{{K}_{m}+C}; \quad f\left(t\right)=\frac{{k}_{a}D{e}^{{-k}_{a}t}}{V};$$

Which yields the following 2 ODEs, Eqs. (5 and 6).

5 $$\frac{{dy}_{1}}{dt}=-{k}_{a}{y}_{1}; \quad {y}_{1}\left({t}_{0}\right)=Dose$$

6 $$V\frac{d{C}^{\left[n\right]}}{dt}={k}_{a}{y}_{1}-\left(\frac{{V}_{max}}{{K}_{m}+{C}^{\left[n-1\right]}\left(t\right)}\right){C}^{\left[n\right]}\left(t\right); \quad { C}^{\left[n=0\right]}\left(t\right)=\frac{{y}_{2}^{\left[n=0\right]}}{V}=0; \quad C\left({t}_{0}\right)=\frac{{y}_{2}\left({t}_{0}\right)}{V}=0;$$

Importantly there are now two types of initial values that need to be described for this solution, the initial values for the plug in $C({t}^{\left[n=0\right]}=0; \forall t)$ and the initial values for the 2 ODEs at $t=0$.

For $n=\mathrm{1,2},3\dots ,N$, using the EVD approach for the matrix exponential solution [9], we generate the solution for the *n*th iterate expressed in Eq. (7). Let the rate constant matrix $K$ for the *n*th iterate of the ODE system be

7 $$\frac{d{{\varvec{y}}}^{[n]}}{dt}=\left[\begin{array}{c}\frac{{dy}_{1}}{dt}\\ \frac{{dy}_{2}^{[n]}}{dt}\end{array}\right]=K\cdot {y}^{\left[n\right]}$$

8 $$K=\left[\begin{array}{cc}{-k}_{a}& 0\\ {k}_{a}& -\frac{1}{V} \frac{{V}_{max}}{{K}_{m}+{C}^{\left[n-1\right]}}\end{array}\right]$$

9 $${C}^{\left[n\right]}\left(t\right)=\frac{1}{V}{{\overline{V} }_{n}e}^{{\lambda }_{n}t}{{\overline{V} }_{n}}^{-1}{k}_{a}D{e}^{{-k}_{a}t} ;$$

where $\overline{V }$ are the eigenvectors of $K$ and $\lambda$ are the eigenvalues. Given the initial values of ${C}^{\left[0\right]}$= 0 then the first iteration of the IndLin is linear as Eq. 8 becomes:

10 $$K=\left[\begin{array}{cc}{-k}_{a}& 0\\ {k}_{a}& -\frac{1}{V}\frac{{V}_{max}}{{K}_{m}}\end{array}\right]$$

and the $K(\mathrm{2,2})$ entry is now constant. The $K$ matrix (Eq. 8) is now LTV for subsequent iterations.

We explore efficiency improvements in the IndLin method coupled with EVD for simulation and estimation in the following two sections. Note that matrix exponentials using EVD provides an exact solution for linear time-invariant systems. We explore its use in LTV in the next section. In all cases, we use *ode45*, the standard non-stiff ODE solver in MATLAB, R2020a (9.8.0.1323502), as our reference solution.

## Improvement in the efficiency of *Inductive Linearization* coupled with EVD for simulation

This section is in two parts. In part 1, we introduce a stopping rule for the maximum number of iterations ($N$) for IndLin and, in part 2, we explore the step size for the EVD integration.

### Part 1: Stopping rule (convergence) for *Inductive Linearization*

As proposed, the IndLin method continues iteratively until $N$ iterations are reached. To make the method more computationally efficient, a stopping rule based on successive errors was constructed with the error compared to some predefined level of tolerance. Here we propose a stopping rule comparing the successive absolute relative error to a predefined tolerance ($\varepsilon$) (Eq. 11).

11 $$max\left(\left|\frac{{y}^{\left[n\right]}(t)-{y}^{\left[n-1\right]}(t)}{{y}^{\left[n\right]}(t)}\right|\right)<\varepsilon ;$$

where |.| denotes the absolute value. In this setting convergence was defined as occurring when the maximum relative error across all time points of interest between two successive iterations $$n$$ and $$n-1$$ was less than $$\varepsilon$$. In this simulation, we compared IndLin without the stopping rule $$(N=60)$$ and IndLin with the stopping rule ($$\varepsilon$$ =$${10}^{-6})$$ to *ode45* (the reference solution). We arbitrarily set the step size for EVD to 0.01 (this is explored in Part 2). The results are shown in Table 2.

**Table 2:** IndLin accuracy and speed without and with the stopping rule compared with the reference solution ode45

Methods	Final maximum absolute relative-error	Run-time (seconds)
*ode45* (reference solver)	–	$0.188$
IndLin $N=60$ without Stopping rule	${3.09e}^{-15}$	$3.921$
IndLin $$N=60$$, with Stopping rule $$(\varepsilon$$ = $${10}^{-6})$$	$$7.12{e}^{-07}$$	$$0.100$$

$$\varepsilon$$: tolerance

As Table 2 shows, applying IndLin without a stopping rule (i.e., where the maximum $N=60$) is considerably slower than the reference solution but is highly accurate. In contrast, the advantage of using a stopping rule allowed convergence to be achieved faster with typically the maximum number of iterations at $N=8$. The solution was now comparable to *ode45* in terms of speed.

### Part 2: Adaptive step size for EVD integration

The EVD solution is exact for linear time-invariant problems. The LTV problem can be divided into a series of time steps over which an exact solution can be calculated, i.e. the step is sufficiently small that the assumption of time invariance is acceptable. The resolution of the step size will affect the speed and accuracy of the approximation to the LTV system. In this part, we consider two fixed step sizes (0.1 and 0.01) and an adaptive step size based on the rate of change in the response variable over time $\left(\frac{dy}{dt}\right)$. For the adaptive method, the step size should be inversely related to the absolute rate of change in response over time such that,

12 $$ss\propto \left|{\left(\frac{dy}{dt}\right)}^{-1}\right|$$

Hence step size will be smaller when the rate of change is highest and vice versa. This yields the expression with a scaling factor of $\alpha$,

13 $$ss=\alpha \left|{\left(\frac{d{y}^{*}}{dt}\right)}^{-1}\right|$$

Since $y$ is a nonlinear function of the parameters and is not available in closed form, the derivative is also not available in closed form. Hence, we approximated ($\frac{dy}{dt}$) with $\frac{\delta y}{\delta t}$(numerically calculated slope) for the first iteration of IndLin, where the plug-in value for ${y}^{*}$ was based on the first run of IndLin. We investigated different combinations of fixed step sizes ($ss=0.01, 0.1$) and adaptive step sizes ($\alpha =0.001,0.01$). The results are shown in Table 3. It is evident that the adaptive step size for EVD was more efficient compared to the fixed step sizes and was comparable to *ode45*. An adaptive step size $\alpha = 0.01$ appeared to provide sufficient resolution.

**Table 3:** Comparison of the IndLin accuracy ($\mathrm{ss}=0.1$, $\mathrm{ss}=0.01$, and adaptive ss using $\mathrm{\alpha }=0.01$) with ode45 for simulation

Methods	Final maximum absolute relative-error	Run-time (seconds)
*ode45* (reference solver)	–	0.188
Fixed step-size^a^		
IndLin $(ss=0.1)$	9.417${e}^{-7}$	0.109
IndLin $(ss=0.01)$	7.123${e}^{-7}$	0.993
Adaptive step-size^a^		
IndLin (adaptive step size $\alpha =0.001$)	${1.69e}^{-7}$	0.421
IndLin (adaptive step size $\alpha =0.01$)	$4.42{e}^{-7}$	0.055

^a^The stopping rule for IndLin was included in these solutions with tolerance ($\varepsilon$) set to 1e^−6^ and maximum number of iterations ($N$) to twenty ($20$)

*ss* step size

In all evaluations of step size the stopping rule described in part 1 of this section was also included.

Although the written scripts for this example state that $\frac{dy}{dt}$, is a vector of the values of $y$ over time using single response measure, this can be generalized to multiple response measures by taking the derivatives of each $y$ variable over time and then keeping the unique values of adaptive step size.

## Improvement in the efficiency of *Inductive Linearization* coupled with EVD for estimation

In this section, we improve the IndLin approach for estimation. A potential advantage of using the *Inductive Linearization* method compared to other solvers is that the IndLin approach can learn from the previous iteration’s values of the parameter vector during the estimation process. During the parameter optimization process (by the nonlinear regression search software) each function evaluation requires the ODE to be linearized using the IndLin-EVD approach. Since IndLin is therefore called many times during the parameter search we can take advantage of the previous linearized solution to initiate the IndLin process. This means that our initial approximation of ${y}_{0}$, instead of being $0$ for all $t$ for each function evaluation of the estimation search, can be replaced by the previous vector of model predicted concentrations from the previous parameter estimates. It is expected that, when a gradient-based search is used, this should provide a more accurate starting position, meaning that the iterative linearization process can be restarted at Eq. 8 rather than Eq. 10. We term this a *smart update* (see the detail below).

**Fig. 1:**
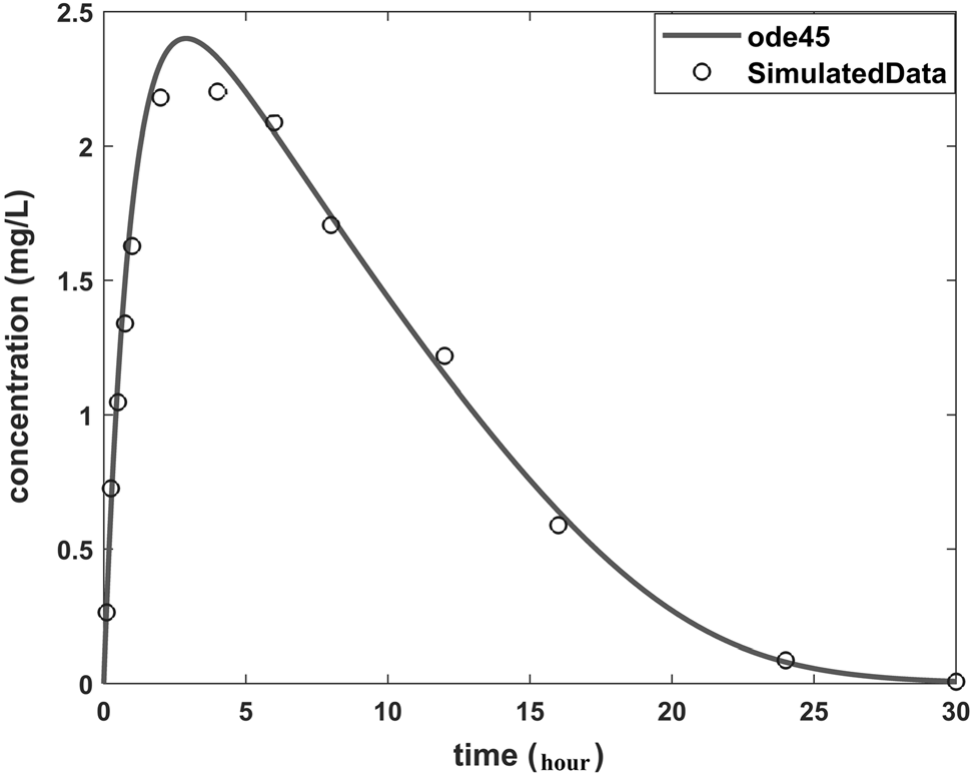
Concentration–time profile for simulated data by typical profile from MATLAB *ode45* and $\sigma =0.1$

Data for a single subject with 13 sampling times were simulated using *ode45* using Eq. 3. The sampling times were $t=[0.1, 0.25, 0.5, 0.75, 1, 2, 4, 6, 8, 12, 16, 24, 30]$ with a dose of $3$ mg and parameter values as per Table 1. An exponential random error structure was applied with standard deviation $\upsigma =0.1$ (i.e. CV = 10%). A typical concentration–time profile together with the simulated data is shown in Fig. 1.

Estimation was based on the *lsqnonlin* function in MATLAB, which uses the gradient based Levenberg–Marquardt (L–M) algorithm. We used the ordinary least squares objective function (Eq. 14) to evaluate the parameter vector $\uptheta =({k}_{a}, {K}_{m}, {V}_{max}, V)$. In this equation the function ($g(\uptheta , {t}_{i}, d)$) represents the model predicted-concentration in the central compartment which was evaluated using either *ode45* or IndLin and $k$ the total number of samples (in this case 13). Importantly inductive linearization was performed around the model predicted concentrations $(g)$ rather than around the data $(y)$, since the latter includes error and hence in Eq. 8${C}_{i}^{\left[n-1\right]}$ is replaced by ${g}_{i}^{\left[n-1\right]}$.

14 $$OFV=\sum_{i=1}^{k}{({y}_{i}-{g}_{i})}^{2}$$

The settings for IndLin included a stopping rule for linearization with $\varepsilon ={10}^{-6}$ and EVD with adaptive step size ($\alpha =0.01$). In this simulation-estimation run the *smart update* was considered where the linearization algorithm was initialized with ${E[y}^{\left[0\right]}(t)]=g({\uptheta }^{\left[\mathrm{m}-1\right]},t,d)$ rather than a vector of $0$ s and $g({\uptheta }^{\left[m-1\right]},t,d)$ represents the model predictions evaluated at the previous parameter vector and ${\uptheta }^{\left[m-1\right]}$ represents the values of the parameter vector at the $m-1$th iteration of the L-M algorithm. For the first iteration of the L–M algorithm (when $m=1$), IndLin was initialized with a vector of $0$ s for all time points.

We see in Table 4 that IndLin with the smart update provides similar parameter estimates to the reference solution *ode45* with a significant speed advantage.

**Table 4:** Comparison of parameter estimates and speed for IndLin and ode45

Parameters	Parameter estimates				Run time (s)
	${k}_{a}(\text{/h}$)	$V ({\text{L}})$	${V}_{max} (\text{mg/h})$	${K}_{m} (\text{mg/L})$	
True parameter values	1	1	0.2	0.5	
*ode45*	$1.20$	$0.94$	$0.25$	$0.42$	$4.24$
IndLin^a^	$1.02$	$0.93$	$0.22$	$0.49$	$29.8$
IndLin, smart update^a^	$1.05$	$0.95$	$0.21$	$0.51$	$1.59$

^a^Tolerance ($\varepsilon$) was set to 1e^−6^ and α = 0.01

## Evaluation of *Inductive Linearization* using stochastic simulation and estimation

In this part, we evaluate the IndLin-EVD improvements from “Improvement in the efficiency of *Inductive Linearization* coupled with EVD for simulation” and “Improvement in the efficiency of *Inductive Linearization* coupled with EVD for estimation” sections for repeated single-subject stochastic simulation estimation (SSE). The objective function, optimization algorithm, dose and sampling times were the same as in “Improvement in the efficiency of *Inductive Linearization* coupled with EVD for estimation” section. No variability in the sampling times or dose were considered.

Assuming that random fluctuations in their parameter values drive the system's dynamics in every individual, the 1000 SSE were performed from the nominal parameter values in Table 1, with added between-subject variability for each parameter (Eq. 15).

15 $${\theta }_{i}=\overline{\theta }\cdot \mathrm{exp}\left({\eta }_{i}\right);$$

where ${\theta }_{i}$ represents the ith patient's parameter (e.g., ${V}_{max, i}$), $\overline{\theta }$ represents the mean parameter (from Table 1), and ${\eta }_{i}$ is the difference of the *i*th subject from the mean. Residual unexplained variability was as per “Improvement in the efficiency of *Inductive Linearization* coupled with EVD for estimation” section (exponnetial error). Note each SSE consisted of only one individual (i.e. this was not a population analysis setting), and hence the estimation model did not include the between-subject variance–covariance matrix ($\Omega$).

IndLin was set up with the same stopping rule as section $3$ and smart update as “Improvement in the efficiency of *Inductive Linearization* coupled with EVD for estimation” section. In addition, the EVD step size was set to both fixed step sizes of $0.1$ and $0.01$ and an adaptive stepsize with $\alpha =0.01$. The initial parameter values for the L–M algorithm were the mean values given in Table 1. The reference solution was provided by *ode45*. The final parameter values, run time (in seconds) and the objective function value were recorded for each estimation and compared among the tested methods. The final parameter values were summarized using the relative difference (%) below:

16 $$\mathrm{RelDiff}=\left(\frac{\widehat{{\theta }_{i}}-{\theta }_{true}}{{\theta }_{true}}\right)\cdot 100$$

**Fig. 2:**
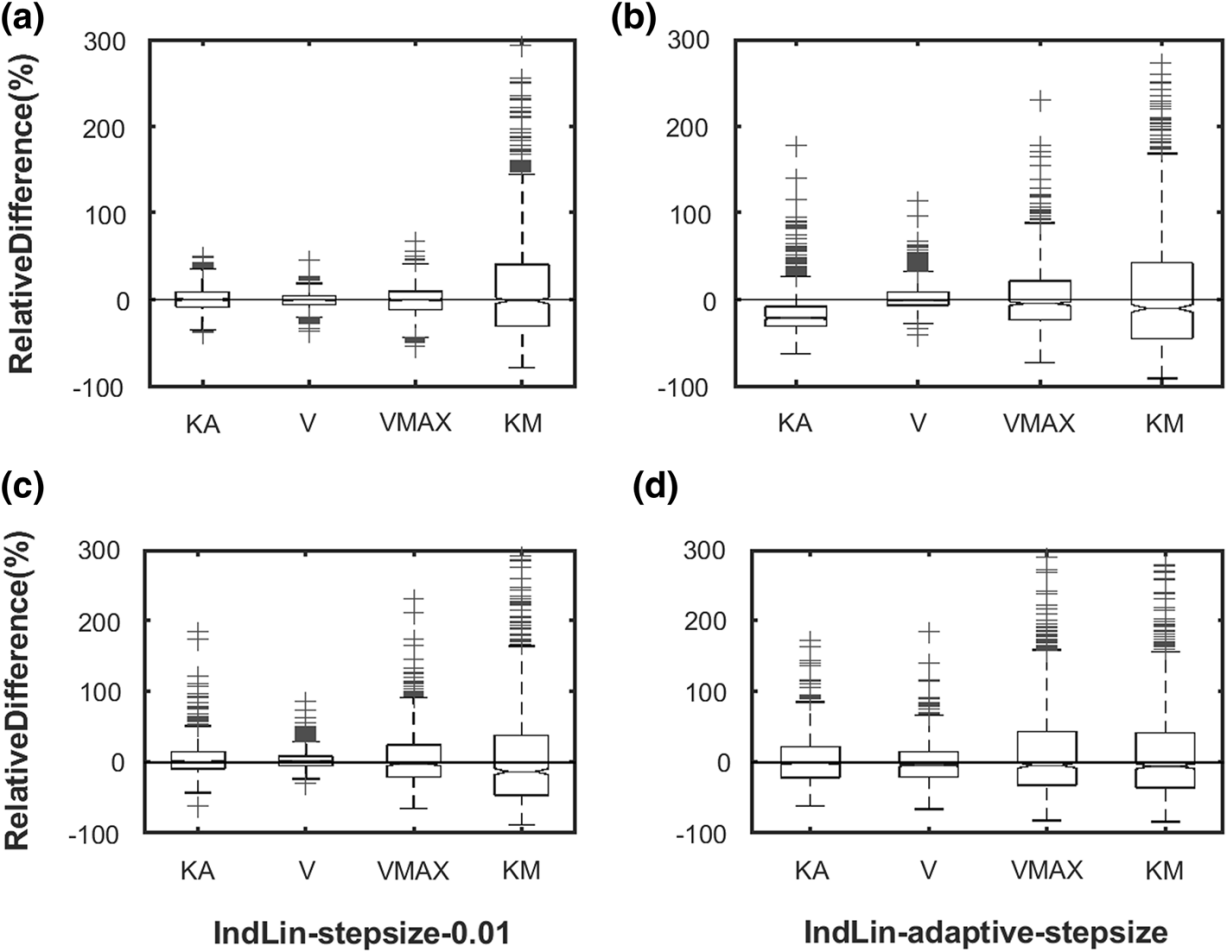
Comparison of relative difference of estimated parameters from their nominal simulation values (%) for **a** ode45, **b** IndLin (ss = 0.1), **c** IndLin (ss = 0.01) and **d** IndLin (adaptive step size, α = 0.01)

**Fig. 3:**
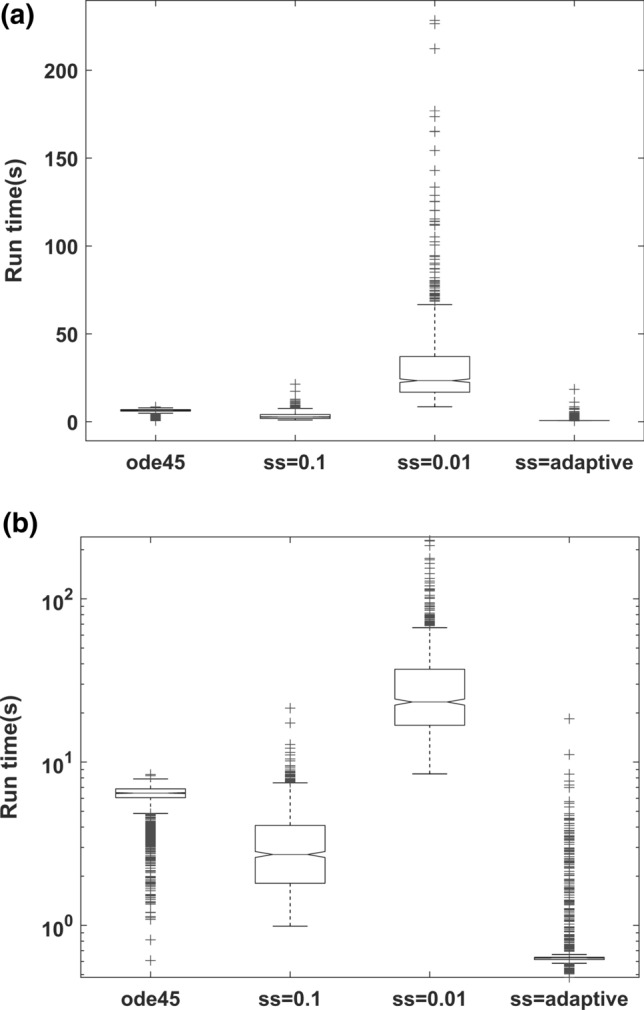
Comparison of speed (seconds) among ode45, IndLin ($ss=0.1$), IndLin ($ss=0.01$) and IndLin (adaptive step size, $\alpha =0.01$) (**a** in linear scale and **b** in log scale)

**Fig. 4:**
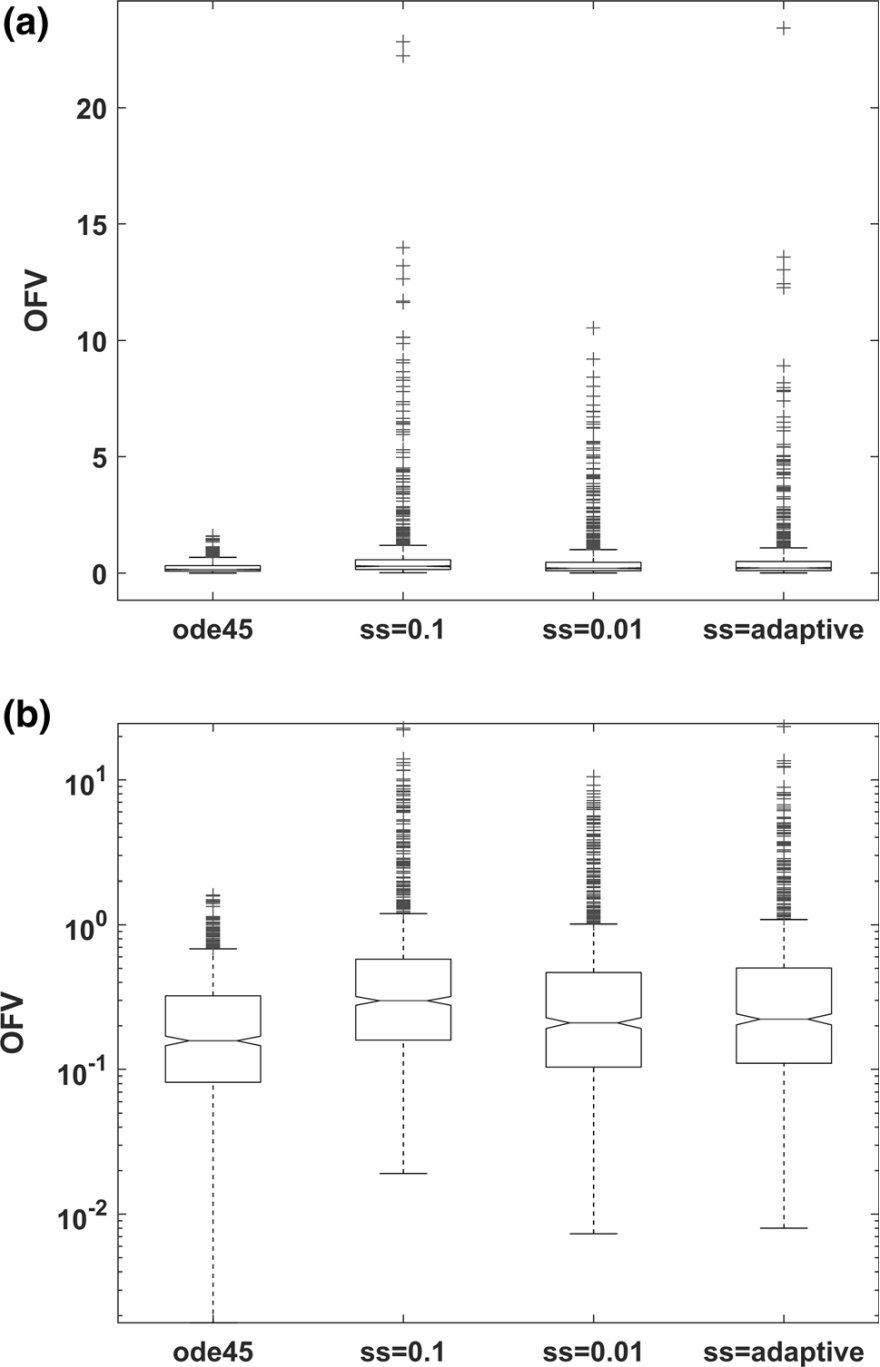
Comparison of the objective function value (OFV) among ode45, IndLin (ss = 0.1), IndLin (ss = 0.01) and IndLin (adaptive step size, α = 0.01) (**a** in linear scale and **b** in log scale)

The relative differences (%) for the parameter estimates are shown in Fig. 2. A similar performance was observed among the reference solution *ode45* (Fig. 2a), IndLin with fixed $ss=0.01$ (Fig. 2c), and IndLin with adaptive step size (Fig. 2d). However, the larger step size of $ss=0.1$ produced an obvious bias in the estimate of ${k}_{a}$ (Fig. 2b). The estimation speed is shown in Fig. 3a (linear scale) and 3b (log scale). The run-time (mean ± standard deviation in seconds) of IndLin when using adaptive step size ($0.857\pm 1.00$) was six times faster than *ode45* ($5.89\pm 1.50$). The speed of IndLin with the fixed $ss=0.1$ ($3.21\pm 1.95$) was faster than that with the fixed $ss=0.01$ ($31.1\pm 24.3$) and slightly faster than *ode45*. The Objective Function Values (OFV) for each run are presented in Fig. 4a (linear scale) and 4b (log scale). For IndLin with fixed $ss=0.1,$ the OFV (mean ± standard deviation) was ($0.768\pm 1.80$), while that with the fixed $ss=0.01$($0.581\pm 1.16$) and that with adaptive step size ($0.637\pm 1.517$) are similar and are slightly larger than that from *ode45* ($0.241\pm 0.242$). Additional information is given in the Supplement (Supplemental, S1). In particular it is shown that OFV is a function of tolerance, and hence reducing tolerance would yield different OFV values. A further set of simulation-estimations with a lower tolerance value were conducted to study this feature, and the findings are included in the Supplement. Since the parameter values were accurate and precise the OFV was considered to be of minor significance.

## Inference and Limitations

In this work, we describe some initial exploration of efficiency improvements of *Inductive Linearization* (IndLin) when coupled with *eigenvalue decomposition* (EVD) to solve a simple system of nonlinear ODEs that is complementary to standard numerical solvers, such as *ode45* (MATLAB). This work builds on the previous descriptions of this algorithm [4, 5] by introducing efficiency improvements of several key steps. Furthermore, the IndLin approach is iterative, and hence introducing an approach that provides an automatic stopping rule for (desired) accuracy and updates its starting conditions provides substantial speed advantages while maintaining accuracy in parameter estimates.

We have not attempted to optimize pairing IndLin with other integrators in this work. Instead, we have considered EVD due to its ease of application. Coupling IndLin with EVD for integration is a pragmatic approach to solving the resulting LTV system; improving its performance with a step size based on the rate of change in the response variable over time provided the expected speed advantages. However, the application of EVD would need to be evaluated for large models, where the number of ODEs greatly exceeds 1000. We note that any integration method that can accommodate LTV systems of ODEs would be appropriate here. This includes the methods based on numerical inverse Laplace transforms, Gaussian quadrature [7], or even a time-stepping ode solver (such as *ode45*). It is important to note that *ode45* is generally faster when used to solve linear problems compared to nonlinear problems (of the comparable dimensionality).

The main difference and potential advantage of the IndLin method vs. numerical time-stepping integrators (e.g., LSODA, Runge–Kutta) is how they solve the problem. Time-stepping ODE solvers generate solutions for the next time-step in the series based on characteristics of their local current solution in a single process [10]. This single process approach makes them very appealing for application to solving ODEs. However, all previous times and future times are unavailable and hidden from the solver. This requires the user to potentially instruct the time-stepping solver about the occurrence of time-based discontinuities that may occur in the function (e.g., the end of an infusion). This contrasts with integrated solutions where the response over the whole time domain is solved simultaneously. The IndLin method lies part way between the time-stepping ODE solver and the fully integrated solution. The linearization process iteratively converts the nonlinear ODE into an LTV ODE system over the whole time span. Hence when using this method, all perturbations to the system that occur at any point in the time span are available and known to the solver and are solved simultaneously. IndLin, however, is a 2 stage process and the LTV ODE still needs to be integrated, and pairing this up with an integrator remains an important step. LTV systems, are often available in the closed-form (see for an example of a closed-form solution for an LTV system [7, 8, 11] are simpler to solve (compared with nonlinear systems) and are defined globally for any legal parameter vector.

In addition, IndLin coupled with EVD integrator could provide an easier computational application than either Gaussian quadrature or Laplace transform to solve the LTV system [7], since EVD could improve the convergence of commonly used numerical analysis such as the Newton algorithm in nonlinear optimization [12]. The Newton direction may not be the descending direction when the Hessian matrix is unfavorable. By applying EVD, the Newton algorithm is modified so that its absolute values replace the negative eigenvalues of the Hessian matrix. Finally, it reconstructs the Hessian matrix and modifies the searching direction.

There are several limitations to this work.

1. The choice of integrator. We used EVD, however eigenvalue decomposition does not exist for all square matrices. Moreover, in matrix algebra, EVD has a computational burden of matrix inversion and multiplication, which makes the method potentially less appealing. Importantly, also, even though we improved the performance of EVD we did not optimize this integration method for the problem.
2. Here, we used a very simple example of a 1-compartment model with first-order input and mixed-order output. The example itself does not pose any real challenges in solving. Extension of the improved method to more complicated systems models and multiple response variables (e.g., insulin-glucose model [13]) remains an important next step. However, we anticipate that the IndLin improvements (stopping rule and smart update) could be generalized to any setting that IndLin could be used (e.g. insulin-glucose [5], a QSP bone model [14]).
3. We did not consider its use in a population analysis setting. This approach cannot currently be implemented in proprietary software such as NONMEM or Monolix or similar. However, potential advantages may occur when solving both the population parameter values and also the individual parameter values using the first-order conditional estimate algorithms of packages such as in NONMEM or nlmixr [15]. Exploring both its use as well as additional layers of optimization need further work.

In this work we have explored efficiency improvements in IndLin-EVD for both simulation and estimation. Other applications to nonlinear ODEs such as proper lumping [16, 17] in which simulation of the response variable is the critical element would also benefit from improvements outlined in “Application of *Inductive Linearization* to the Michaelis–Menten example” section.

## Conclusions

We have explored some improvements in the efficiency of approaches for IndLin when coupled with EVD for solving nonlinear ODEs. The convergence error and updating the initial conditions for estimation proved valuable for speed and accuracy. Further work is needed to establish the place of this method in pharmacometrics.

## Supplementary Information

Below is the link to the electronic supplementary material.

Supplementary file1 (DOCX 74 kb)
