# Supplementary material for: Exploring Inductive Linearization for simulation and estimation with an application to the Michaelis–Menten model
Source: J Pharmacokinet Pharmacodyn. 2022 Jul 5;49(4):445–53. doi: 10.1007/s10928-022-09813-z (PMC9338916; doi:10.1007/s10928-022-09813-z)

**Exploring *Inductive Linearization* for simulation and estimation with an application to the Michaelis-Menten model**

Sepideh Sharif*, Chihiro Hasegawa, Stephen B. Duffull

Otago Pharmacometrics Group, School of Pharmacy, University of Otago, Dunedin, New Zealand

*Corresponding author

Email: sepi.sharif@postgrad.otago.ac.nz

**Supplement**

In this supplement the relationship between the objective function value (OFV) and the tolerance ($\varepsilon$) of its iterative solution is explored for a single simulation-estimation of a single subject.

It was seen that the OFV for IndLin was on average higher than for *ode45*. For a single simulation-estimation for 1 subject the OFV is shown in Table S1 for *ode45*, IndLin with tolerance ($\varepsilon= 1e-6$) with adaptive step size for EVD and with the smart update method. For both settings for IndLin the OFV value was larger than for *ode45*. Estimation for this data set was re-performed for IndLin with various values of tolerance ($\varepsilon$) with the same settings of adaptive step size and smart update. The results are shown in Fig. S1. It is seen that the accuracy, in terms of OFV, relates to the tolerance of the convergence of the IndLin method and achieves a value that is comparable to *ode45*.

Comparison for a simulation estimation example of 50 runs with the lower tolerance ($1e-7$) yielded run time (seconds) of $2.1\pm1.2$ for IL and $6.1\pm1.52$ for *ode45,* indicating that the optimized IndLin with a projected equivalent objective function value retains a speed advantage.

Table S1: Comparison of OFV for IndLin and ode45

| **Methods** | **OFV** |
| --- | --- |
| *ode45* | 0.132 |
| IndLin^1)^ | 0.269 |
| IndLin, smart update^1)^ | 0.294 |

1) For applying stopping rule in *Inductive Linearization*, tolerance ($\varepsilon$) was set to 1e-6 and maximum number of iterations ($N$) and α for adaptive step size to twenty ($20$) and 0.01, respectively.

**Fig. S1**: The relationship between OFV and tolerance (ε) shows OFV is a function of tol


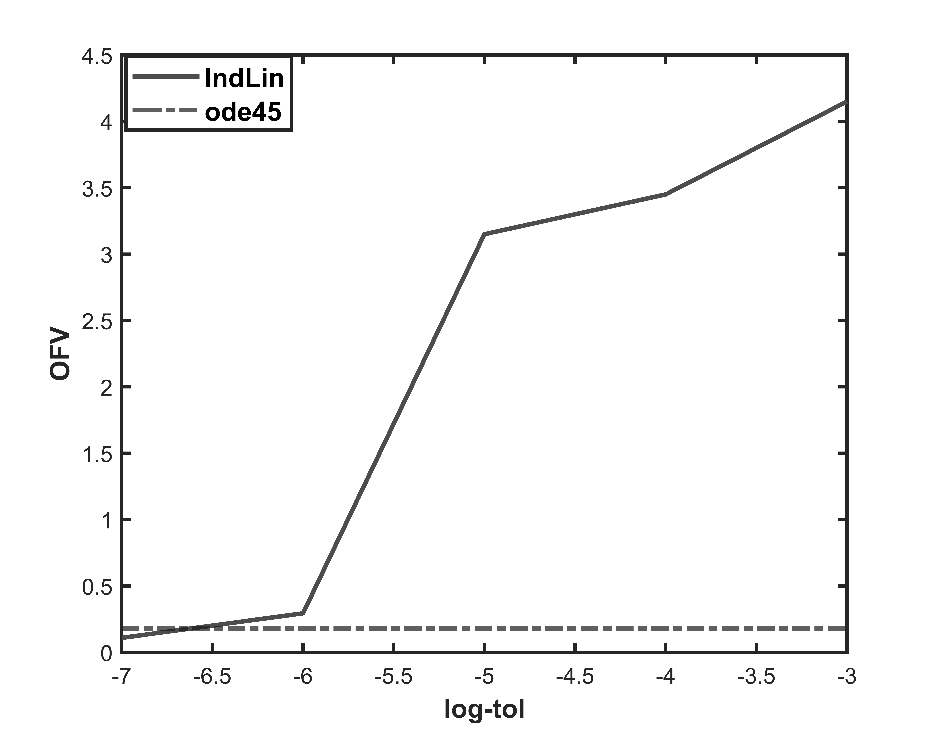

Supplement: Supplementary file 1 — Supplementary file1 (DOCX 74 kb) [file 10928_2022_9813_MOESM1_ESM.docx]
